# IL-33 Participates in the Development of Esophageal Adenocarcinoma

**DOI:** 10.3389/pore.2022.1610474

**Published:** 2022-08-30

**Authors:** Jia Liu, Lei Liu, Yang Su, Yi Wang, Yuchun Zhu, Xiaobin Sun, Yuanbiao Guo, Jing Shan

**Affiliations:** ^1^ School of Medicine, Southwest Jiaotong University, Chengdu, China; ^2^ The Affiliated Hospital of Southwest Jiaotong University, The Third People’s Hospital of Chengdu, Chengdu, China; ^3^ Medical Research Center, The Affiliated Hospital of Southwest Jiaotong University, The Third People’s Hospital of Chengdu, Chengdu, China; ^4^ North Sichuan Medical College, Nanchong, China; ^5^ Department of Gastroenterology, The Affiliated Hospital of Southwest Jiaotong University, The Third People’s Hospital of Chengdu, Chengdu, China

**Keywords:** EMT, esophageal adenocarcinoma, interleukin-33, ST2, EAC rat model

## Abstract

**Background:** The progression from chronic gastroesophageal reflux disease (GERD) to Barrett esophagus (BE) and esophageal adenocarcinoma (EAC) is an inflammatory-driven neoplastic change. Interleukin-33 (IL-33) has identified as a crucial factor in several inflammatory disorders and malignancies.

**Methods:** The high-density tissue microarray of the human EAC was analyzed with IL-33 immunohistochemistry staining (IHC). By anastomosing the jejunum with the esophagus, the rat model of EAC with mixed gastroduodenal reflux was established. The expression of IL-33 was determined using quantitative real-time polymerase chain reaction (RT-qPCR), western blot (WB), IHC and enzyme-linked immunosorbent assay (ELISA). Esophageal adenocarcinoma cells (OE19 and OE33) and human esophageal epithelial cells (HEECs) were used.

**Results:** In the cytoplasm of human EAC tissue, IL-33 expression was substantially greater than in adjacent normal tissue. In rat model, the expression of IL-33 in the EAC group was considerably greater than in the control group, and this expression increased with the upgrade of pathological stage. In *in vitro* experiment, the mRNA and protein levels of IL-33 were considerably greater in OE19 and OE33 than in HEECs. The stimulation of IL-33 enhanced the proliferation, migration, invasion, and epithelial-mesenchymal transition (EMT) of OE19 and OE33, but soluble ST2 (sST2) inhibited these effects. IL-33 stimulated the release of IL-6 by OE19 and OE33 cells.

**Conclusion:** This study demonstrated the overexpression of IL-33 in the transition from GERD to EAC and that IL-33 promoted carcinogenesis in EAC cells through ST2. IL-33 might be a possible preventive target for EAC.

## Introduction

In western countries, the frequency of EAC has increased more than sixfold during the past 4 decades (1). Due to the late presentation of symptoms and the limitation of endoscopic surveillance, EAC is frequently associated with late diagnosis, poor prognosis, significant morbidities, and high mortality rates (2, 3). The development of EAC follows an inflammatory-driven neoplastic transformation, with chronic GERD and BE being classified as major risk factors for EAC development (4, 5).

IL-33 is a relatively new tissue-derived cytokine that is a novel member of the IL-1 cytokine family (6). IL-33 is constitutively expressed in endothelial and epithelial cells, which uniquely functions as a cytokine and a nuclear factor. The cytokine IL-33 performs biological functions by binding to and activating its receptor ST2, which belongs to the Toll-like receptor superfamily (7). Both innate and adaptive immunity are regulated by epithelial-derived IL-33. IL-33 is implicated in tumor-associated inflammation, such as ulcerative colitis, gastritis and GERD (8-10). Furthermore, IL-33 levels are elevated in a variety of cancers, including gastric cancer, colon cancer, and head and neck cancer (11-13). And it has been shown to promote tumorigenesis and facilitate metastasis in several cancer models (14-16). Nonetheless, the biological function of IL-33 in human esophageal adenocarcinoma remains unknown.

Since IL-33 has been proven to facilitate inflammation in GERD (17), we investigated the function of IL-33/ST2 signaling in the development of esophageal adenocarcinoma. In this study, we have revealed for the first time that IL-33 was involved in the inflammatory-driven neoplastic transformation of EAC. Through the IL-33/ST2 pathway, IL-33 increased the proliferation, migration, and invasion of EAC cells. Our findings indicate that IL-33 plays a crucial role in EAC development and might be a therapeutic target.

## Materials and Methods

### Tissue Microarray

Shanghai Biochip Company provided the human EAC high density tissue microarray (HGEj-Ade130Sur-01), which includes primary tumor specimens and matched adjacent tissues from 65 EAC patients. Between September 2006 and December 2009, all patients had EAC resection. Preoperative chemotherapy or radiotherapy was not given to any of these patients. All cases were pathologically confirmed. The tumor-node-metastasis (TNM) stage was determined using the 7th version of the American Joint Committee on Cancer Criteria (AJCC).

### Immunohistochemistry

The tissues were fixed in 10% formalin at 4°C for 24 h before being embedded in paraffin. Tissue samples were sectioned into 4 µm-thick slices, and paraffin-embedded slices were dewaxed in dimethylbenzene and rehydrated in a graded ethanol solution. Antigen retrieval was carried out in citrate solution (10 mmol/L, pH 6.0) at 100°C for 30 min. After 15 min in 0.3% hydrogen peroxide solution to block endogenous peroxidase activity, tissues were incubated overnight at 4°C with the polyclonal rabbit anti-IL-33 (1:2,000; MBL Co., Ltd., Nagoya, Japan for human tissue; 1:200; Proteintech Group, Inc., Chicago, IL for rat tissue). The tissues were then incubated for 30 min at 37°C with the horseradish peroxidase (HRP) labeled goat anti-mouse/rabbit IgG polymer (1:5,000; Fuzhou Maixin Biotechnology Co., Ltd.). Color development was carried out using the enhanced DAB chromogenic kit (Fuzhou Maixin Biotech Co., Ltd.). After counterstaining at room temperature for 5 min with hematoxylin, the samples were dehydrated in a gradient of ethanol and fixed with neutral resin. The slides were then observed and photographed using a light microscope. The IL-33 immunostaining score was calculated by multiplying the intensity score by the positive rate score. The intensity of staining was measured as follows: 1) 0, no staining; 2) 1, weak staining; 3) 2, moderate staining; and 4) 3, strong staining. The positive staining cell rate was measured as follows: 1) 0, 0%–5%; 2) 1, 5%–25%; 3) 2, 26%–50%; 4) 3, 51%–75%; and 5) 4, >75%.

### Construction of Esophageal Adenocarcinoma Rat Model

Wistar rats aged 5 weeks, weighing 220–300 g, were selected to establish the esophageal adenocarcinoma model of gastroduodenal mixed reflux by anastomosing the jejunum with the esophagus, following the established method (18). A 2 cm incision was performed in the upper abdomen to separate the connective tissue between the liver and stomach, retain the vagus nerves on both sides of the esophagus, separate the abdominal esophagus form the cardia, and cut off and suture the distal end. The lower esophagus was intermittently sutured with the distal ileum of Treitz ligament end to side with 7/0 silk thread. The rats in the control group were also anesthetized, opened, and separated, but not anastomosed. 50 mg/kg iron dextran was administered intramuscularly every 4 weeks postoperatively. The rats in the control group were killed 4 weeks after surgery, and the rats in the surgery group were killed 4, 10, and 16 weeks after surgery. After cardiac blood collection, the esophagus and the proximal ileal segment from the anastomosis (0.5 cm) were completely removed in each group. Excluding the dead rats, there were 10 rats in the control group, 8 rats in the 4w group, 9 rats in the 10w and 16w groups. Low-grade dysplasia occurred in all rats in the 4w group, 1 rat in the 10w group, and 3 rats in the 16w group. High-grade dysplasia occurred in 7 rats in the 10w group and 2 rats in the 16w group; EAC occurred in 1 rat in the 10w group and 4 rats in the 16w group.

### Quantitative RT-PCR

Total RNA was extracted from tissue using TRIzol^®^ reagent (Invitrogen; Life Technologies, Inc.). The obtained cDNA was subjected to RT-qPCR using TB Green®Premix Ex Taq™ (Takara Bio, Inc.) on the LightCycler/LightCycler 480 System (Roche, Inc.) after the contaminated DNA was removed and reverse transcribed using Primescript™ RT Reagent Kit with gDNA Eraser (Takara Bio, Inc.). GAPDH was used as internal reference. Each sample was performed in triplicate, and relative RNA levels were calculated using the 2^−ΔΔCq^ method. The sequences were as follows: IL-1β: Forward: 5′-AAT​CTC​ACA​GCA​GCA​TCT​CGA​CAA​G-3′ and reverse: 5′- TCC​ACG​GGC​AAG​ACA​TAG​GTA​GC-3′; IL-4: Forward: 5′- CAA​GGA​ACA​CCA​CGG​AGA​ACG​AG-3′ and reverse: 5′- TTC​TTC​AAG​CAC​GGA​GGT​ACA​TCA​C-3′; IL-6: Forward: 5′- CCG​CAA​GAG​ACT​TCC​AGC​CAG​TTG-3′ and reverse: 5′- CGG​AAC​TCC​AGA​AGA​CCA​GAG​CAG​A-3′; IL-10: Forward: 5′- GGC​AGT​GGA​GCA​GGT​GAA​GAA​TG-3′ and reverse: 5′- TGT​CAC​GTA​GGC​TTC​TAT​GCA​GTT​G-3′; IL-33: Forward: 5′-GAA​CCC​GCC​AAA​AGA​TAT​TCA​C-3′ and reverse: 5′-AAG​TTC​CTT​GGA​TAC​TCA​GTG​TG-3′; GAPDH: Forward: 5′-CCA​TCA​ACG​ACC​CCT​TCA​TT-3′ and reverse: 5′-GAC​CAG​CTT​CCC​ATT​CTC​AG-3′; ST2: Forward: 5′-CGT​TAC​CTT​CCT​GTG​CCA​TT-3′ and reverse: 5′-CTC​CAT​TTG​CCA​ATC​ATG​TG-3′.

### Cell Line and Cell Culture

ATCC supplied the human esophageal adenocarcinoma cell lines (OE19 and OE33), as well as human normal esophageal epithelial cells (HEECs). OE19 and OE33 were cultured in RPMI-1640 medium (Gibco, Grand Island, NY, United States), while HEECs were cultured in Dulbecco’s modified Eagle’s medium (DMEM; Hyclone; Cytiva), supplemented with 10% fetal bovine serum (Gibco, Grand Island, NY, United States), L-glutamine, 100 U/ml penicillin, and 0.1 mg/ml streptomycin (SigmaAldrich; Merck KGaA) at 37°C, 5% CO_2_ air atmosphere.

### Cell Proliferation Assay

The effect of IL-33 on the proliferation of OE19 and OE33 cells was assessed using the CCK8 assay. The cells were seeded at a density of 2,000 cells per well in a 96-well plate. After 24 h, the cells were incubated with IL-33 (Proteintech Group, Inc., Chicago, IL, United States) in increasing concentrations (0, 20 and 50 ng/ml) for 24 h, 48 h and 72 h, respectively. We used sST2 to block the IL-33/ST2 pathway (200 ng/ml) (Immune Technology Corp, NY, United States), which was administered 24 h before adding IL-33. The cells were then washed with PBS and incubated for 3 h with 100 µl of CCK8 solution. The multifunctional fluorescence microplate reader (Polarstar Otima, BMG, Australia) was then used to measure the optical density (OD) at 450 nm.

### Cell Migration Assay

The capacity for cell migration was evaluated using the wound-healing experiment. In 12-well flat-bottomed plates, cells were planted to attain 80%–90% monolayer confluence. A straight line was scratched in the middle of the well with the tip of a sterile pipette. The wound margins of different treatment groups were inspected and photographed after washing the cell debris and administering 50 ng/ml IL-33 after 24 and 36 h. The migration rate was reported as the proportion of the average migration distance to the average initial (0 h) wound distance.

### Cell Invasion Assay

The invasive experiment was conducted utilizing a Matrigel-coated 24-well Transwell (BD Biosciences, San Jose, CA, United States) (8-m pore size; Corning, NY, United States). The upper chamber has 2 * 10^5^ cells suspended in 200 µl of serum-free media with PBS, 50 ng/ml IL-33, 200 ng/ml sST2 and 50 ng/ml IL-33 + 200 ng/ml sST2; The lower chamber contains 500 µl of medium supplemented with 10% FBS. After 24 h, cotton swabs were used to remove the cells on the upper surface of the membrane. The lower surface cells were subsequently fixed with paraformaldehyde and stained with crystal violet. Finally, invasive cells were enumerated and collected in five microscopic fields.

### Western Blot Analysis

The whole cell proteins were extracted by RIPA lysis buffer. After being boiled for 5 min, protein samples were separated by 10% SDS-PAGE, transferred to PVDF membranes, and incubated overnight at 4°C with primary antibodies against IL-33 (1:1,000, Proteintech Group, Inc., Chicago, IL, United States), E-cadherin (1:25,000, Proteintech Group, Inc., Chicago, IL, United States) and N-cadherin (1:1,000, Abcam, Cambridge, United Kingdom). The next day, the membranes were washed and incubated with the HRP-conjugated secondary antibody (1:5,000, Proteintech Group, Inc., Chicago, IL, United States) for 2 h at room temperature. GAPDH (1:2,000, Proteintech Group, Inc., Chicago, IL, United States) was used as internal reference.

### Measurement of Cytokines

Enzyme-linked immunosorbent assay (ELISA) for IL-33 and sST2 in serum was performed using the IL-33 Quantikine ELISA Kit (R&D Systems, Inc.) and the sST2 Quantikine ELISA Kit (R&D Systems, Inc.). The supernatant of the cells was collected and centrifuged at 4°C at 1,000 g for 10 min. IL-4 Quantikine ELISA kit (R&D Systems, Inc.) and IL-6 Quantikine ELISA kit (R&D Systems, Inc.) were used to measure the protein secretion of IL-4 and IL-6 in esophageal adenocarcinoma cells following the manufacturer’s instructions.

### Statistical Analysis

All data analysis was performed as the mean ± SD using SPSS 22.0 (SPSS, Inc.). All experiments were repeated at least three times. Data was analyzed using unpaired t-tests for two groups and one-way ANOVA followed by Scheffe’s F test for multiple comparisons. All tests were two-sided with a significance level of *p* < 0.05.

## Results

### IL-33 Was Upregulated in the Cytoplasm in Human EAC

**FIGURE 1:**
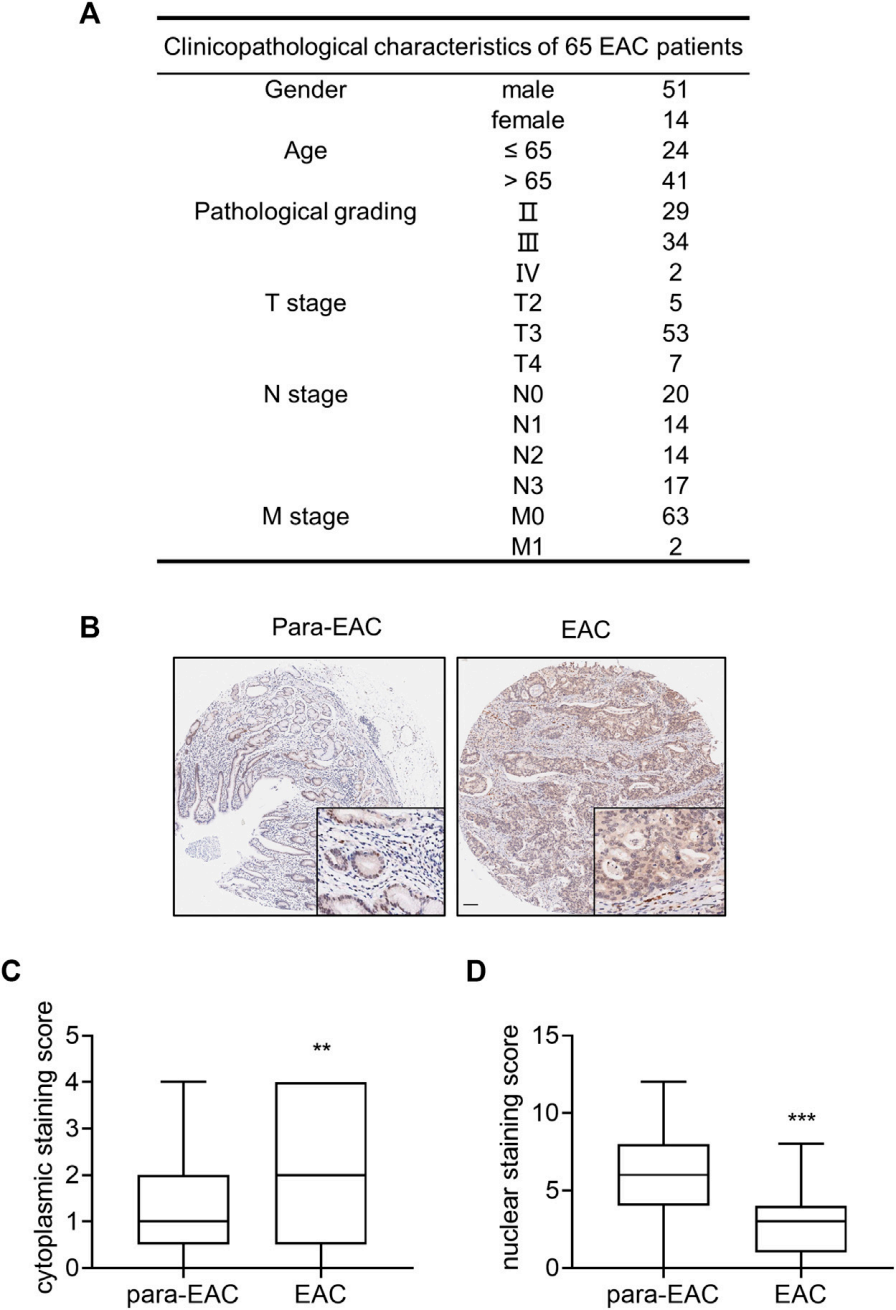
The expression of IL-33 in tissue microarray of EAC. Tissue microarray of EAC in 65 cases was used to analyze the expression of IL-33. **(A)** The clinicopathological parameters of 65 cases included in the tissue microarray. **(B)** The representative images of IL-33 IHC staining in adjacent tissue (para-EAC) and EAC. **(C)** The staining scores of cytoplasmic IL-33 in EAC and adjacent tissues. **(D)** The staining scores of nuclear IL-33 in EAC and adjacent tissues. **: *p* < 0.01, ***: *p* < 0.001.

To investigate the expression of IL-33 in EAC, a high-density tissue microarray containing 65 pairs of EAC and surrounding tissue was utilized (Figure 1A). IHC showed that IL-33 was mainly expressed in the cytoplasm in EAC cells and in the nucleus of adjacent normal epithelial cells (Figure 1B). The staining scores revealed that EAC cells had considerably greater cytoplasmic expression of IL-33 than adjacent normal epithelial cells (Figure 1C), but significantly lower nucleus expression of IL-33 than adjacent normal epithelial cells (Figure 1D).

### IL-33 Was Upregulated With the Upgrade of Pathological Stage From Inflammation to Cancer

**FIGURE 2:**
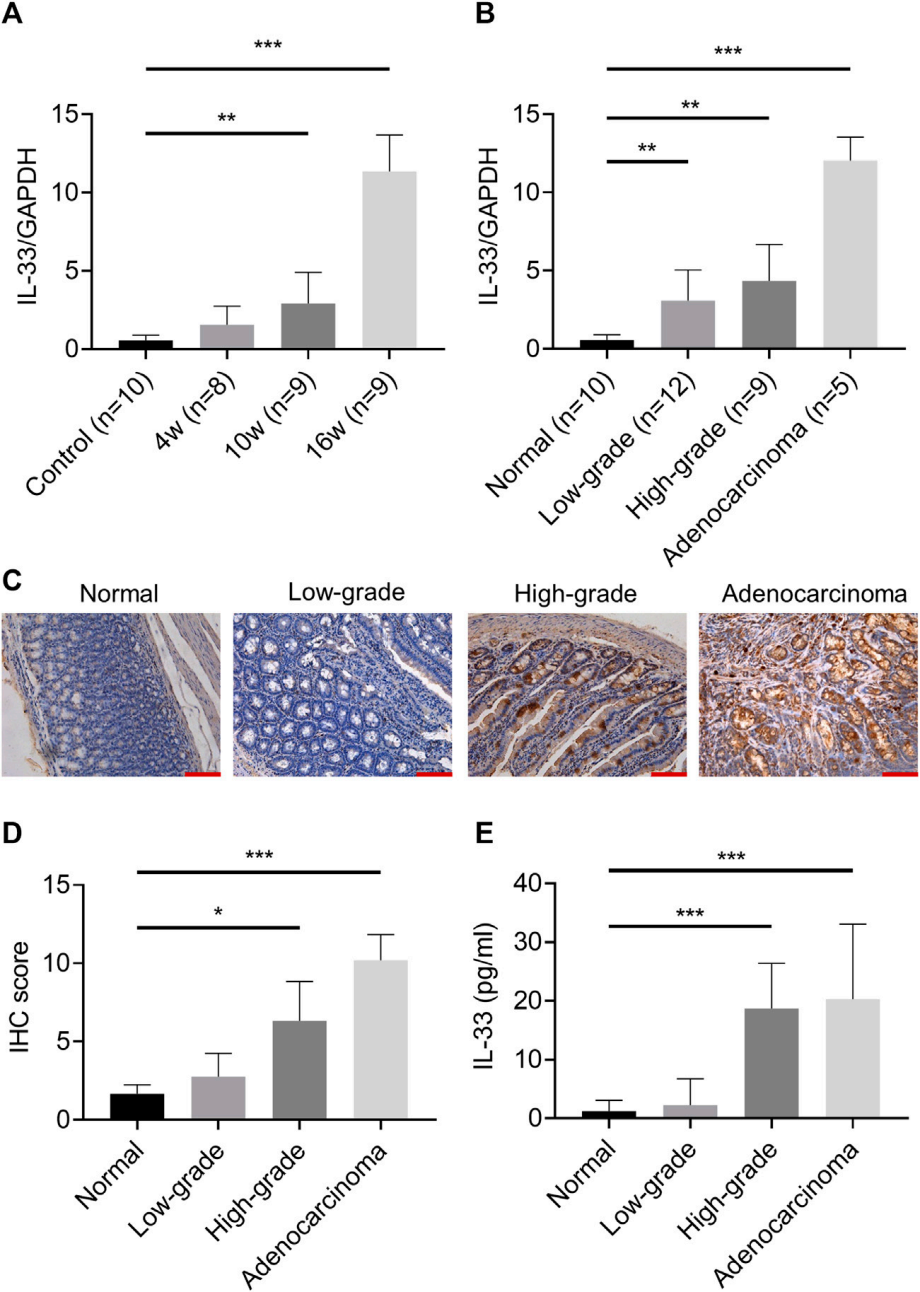
The expression of IL-33 in esophageal adenocarcinoma rat model. The mRNA expression of IL-33 was determined by RT-qPCR in different groups **(A)** and in different pathological stages **(B)** in esophageal adenocarcinoma rat model. **(C)** The representative images of IL-33 IHC staining in different pathological stages. **(D)** The IL-33 staining score in different pathological stages. **(E)** The level of IL-33 in serum was determined by ELISA. Bar: 100 μm *p < 0.05, **p < 0.01, ***p < 0.001. (Low-grade: low-grade dysplasia group; High-grade: high-grade dysplasia group).

In the esophageal adenocarcinoma rat model, the mRNA expression of IL-33 was significantly higher in the 10w and 16w groups than that in other groups (Figure 2A), and increased with the upgrade of pathological stage (Figure 2B). IHC showed that IL-33 was mainly expressed in the cytoplasm in esophageal adenocarcinoma cells (Figures 2C,D). The content of IL-33 in serum also increased with the upgrade of pathological stage (Figure 2E). However, the content of sST2 is too low to be detected.

### IL-33 Expression Increased in Esophageal Adenocarcinoma Cell Lines

**FIGURE 3:**
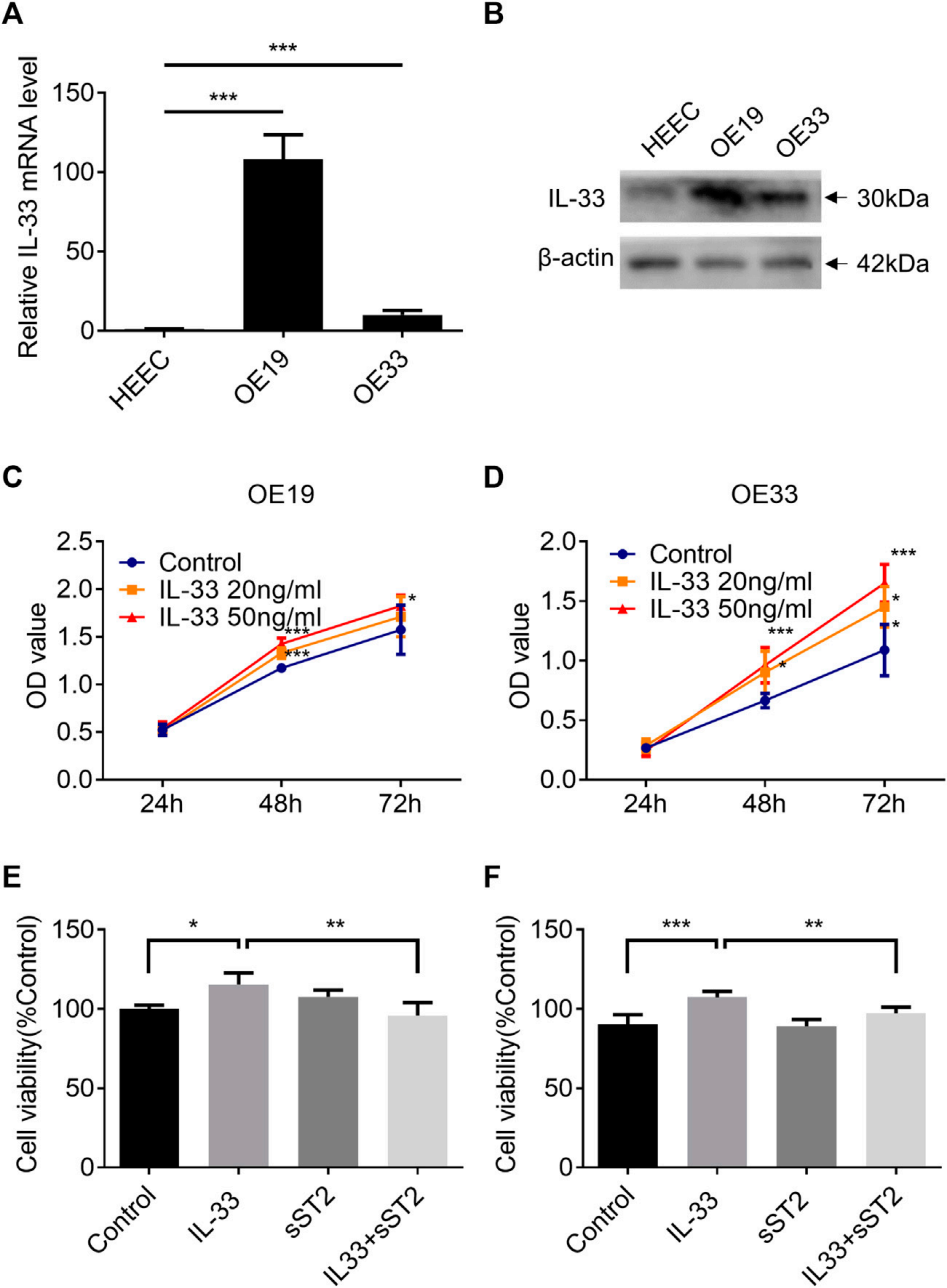
IL-33 promote EAC cell proliferation through ST2. The mRNA expression **(A)** and protein level **(B)** of IL-33 in esophageal adenocarcinoma cell lines (OE19, OE33) and normal esophageal epithelial cells (HEECs) were measured by RT-qPCR and western blotting. The proliferation of OE19 **(C)** and OE33 **(D)** was increased after being stimulated by IL-33 dose and time dependently. After preincubation with sST2 (200 ng/ml), followed by IL-33 (50 ng/ml 48 h) treatment can antagonize the effect of IL-33 on the proliferation of OE19 **(E)** and OE33 **(F)**. Each value represents the mean ± SD of three independent experiments. *p < 0.05, **p < 0.01, ***p < 0.001.

Two esophageal adenocarcinoma cell lines (OE33: poorly differentiated adenocarcinoma; OE19: moderately differentiated adenocarcinoma) and human normal esophageal cells (HEECs) were used to detect IL-33. OE19 and OE33 cells have substantially greater IL-33 mRNA and protein levels than HEECs. (Figures 3A,B).

### IL-33 Promoted Cell Proliferation of Esophageal Adenocarcinoma *via* Its Receptor ST2

The CCK8 assay demonstrated that IL-33 increased the proliferation of OE19 (Figure 3C) and OE33 (Figure 3D) in a time- and dose-dependent manner. Blocking the IL-33/ST2 pathway with sST2 could attenuate the effect of IL-33 on the proliferation of OE19 (Figure 3E) and OE33 (Figure 3F).

### IL-33 Promoted Cell Migration, Invasion and EMT *via* Its Receptor ST2

**FIGURE 4:**
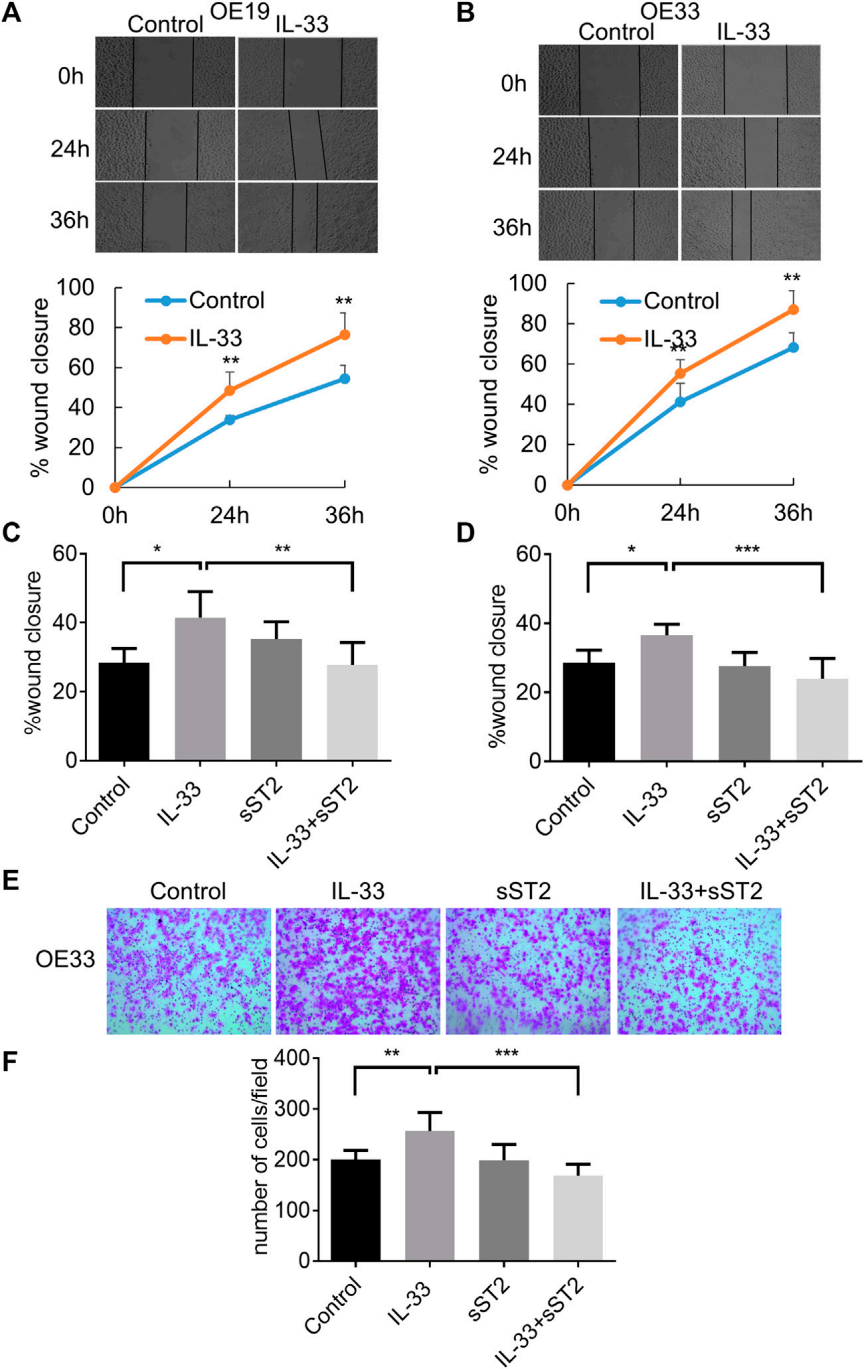
IL-33 promoted EAC cell migration and invasion through ST2. The migration of OE19 **(A)** and OE33 **(B)** was detected by wound healing assay. Scratch healing was photographed under a microscope after 0, 24 and 36 h. After preincubation with sST2 (200 ng/ml), followed by IL-33 (50 ng/ml 24 h) treatment can antagonize the effect of IL-33 on the migration of OE19 **(C)** and OE33 **(D)**. **(E,F)** Transwell assay for OE33 with control, IL-33, sST2 and IL-33+sST2 groups. Each value represents the mean ± SD of three independent experiments. *p < 0.05, **p < 0.01, ***p < 0.001.

In the wound healing assay, IL-33 greatly increased the migration of OE19 (Figure 4A) and OE33 (Figure 4B), which can be attenuated by pretreatment with sST2 (Figures 4C,D). As OE19 cannot pass through the Matrigel transwell, we only detected the effect of IL-33 in OE33 for the invasion assay. We found that IL-33 considerably enhanced the invasion capacity of OE33, and this effect could be weakened by sST2 (Figures 4E,F).

**FIGURE 5:**
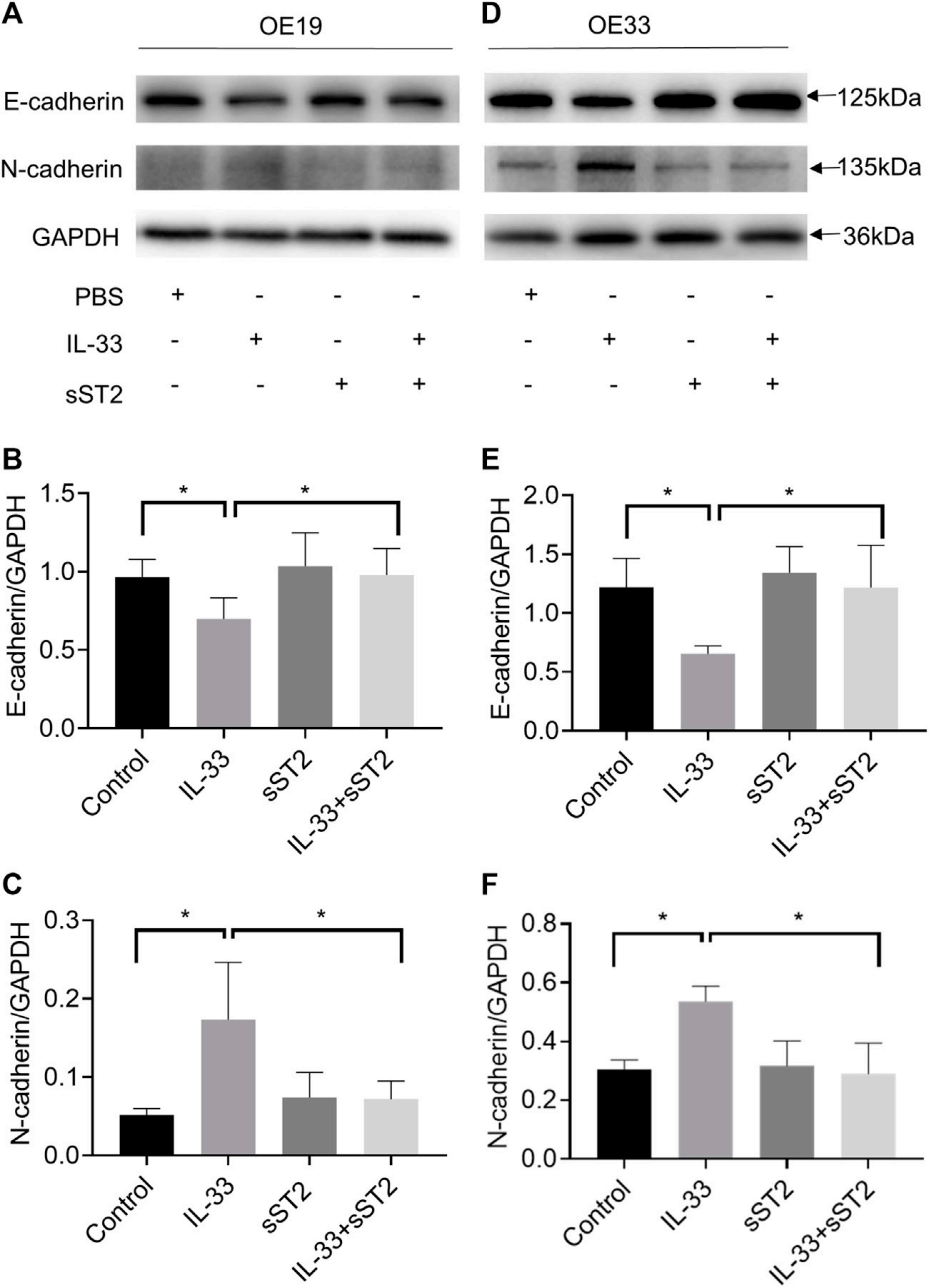
IL-33 promoted epithelial-mesenchymal transition (EMT) in esophageal adenocarcinoma cells. The protein levels of the EMT markers, E-cadherin and N-cadherin were detected by Western blot in OE19 **(A)** and OE33 **(D)** with control, IL-33, sST2 and IL-33+sST2 groups. Western blot analysis for E-cadherin and N-cadherin expression in OE19 **(B-C)** and OE33 **(E-F)**. Each value represents the mean ± SD of three independent experiments. *p < 0.05, **p < 0.01.

We investigated further the function of IL-33 in EMT, which plays a crucial role in tumor migration and invasion. OE19 and OE33 were stimulated with IL-33 (50 ng/ml) for 12 h to investigate the effect of IL-33 on the expression of EMT markers. In OE19 (Figures 5A–C) and OE33 (Figures 5D–F), IL-33 dramatically decreased E-cadherin expression while increasing N-cadherin expression, which could be inhibited by pretreatment with sST2.

### IL-33 Promoted the Secretion of IL-6

**FIGURE 6:**
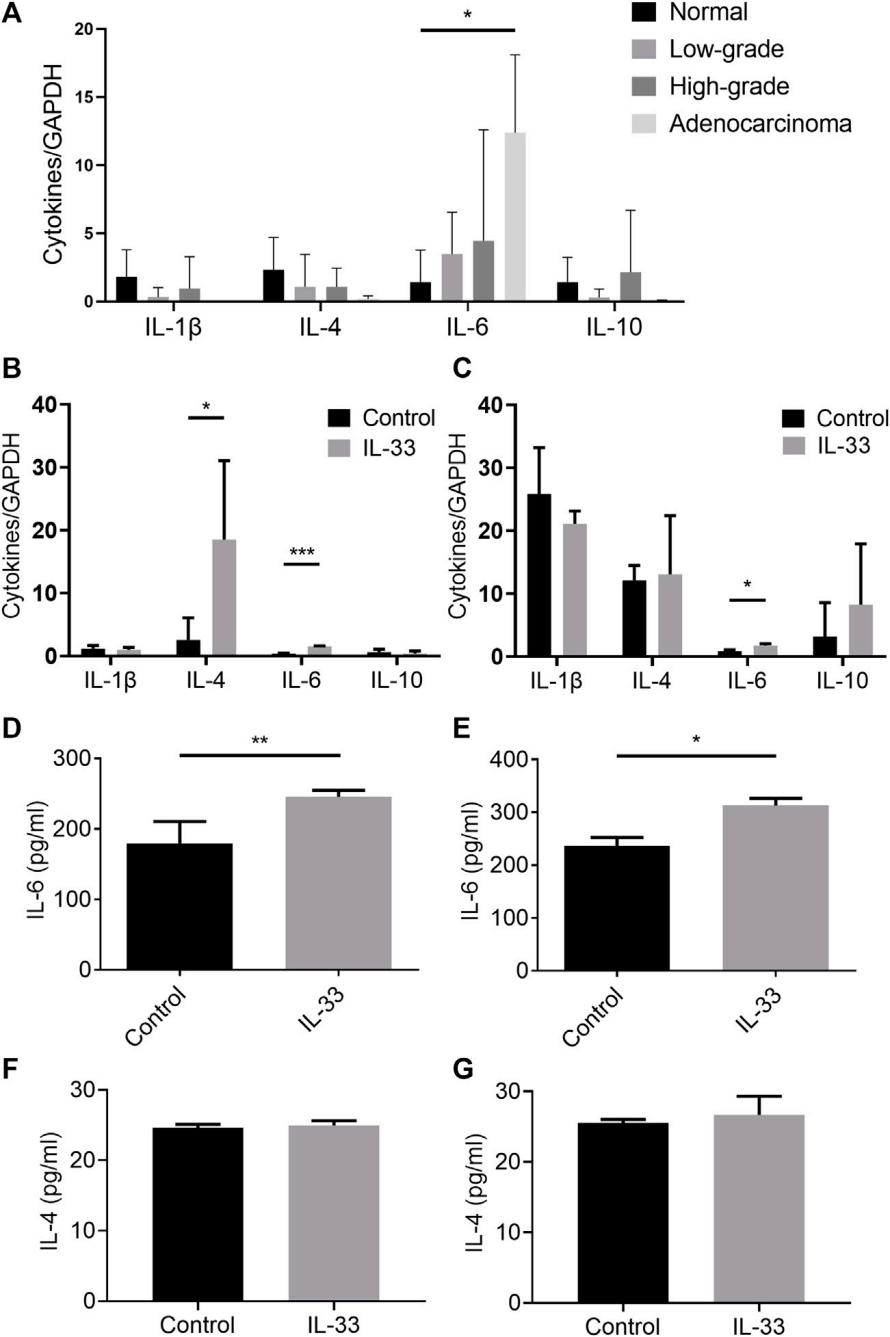
IL-33 promoted the secretion of IL-6. The tissue mRNA expressions of IL-1β, IL-4, IL-6, and IL-10 were determined by RT-qPCR in different pathological stages in esophageal adenocarcinoma rat model **(A)**. The cell mRNA expressions of IL-1β, IL-4, IL-6 and IL-10 were determined by RT-qPCR in IL-33 stimulated cells OE19 **(B)** and OE33 **(C)**. The levels of IL-4 and IL-6 in IL-33 stimulated cells OE19 **(D,F)** and OE33 **(E,G)** were detected by ELISA. Each value represents the mean ± SD of three independent experiments. *p < 0.05, **p < 0.01, ***p < 0.001.

We detected the tissue mRNA expression of IL-1β, IL-4, IL-6, and IL-10 in the esophageal adenocarcinoma rat model (Figure 6A). The mRNA expression of IL-6 was significantly higher in the adenocarcinoma group than that in the normal group (Figure 6A). However, we did not detect the alteration of IL-6 content in rat model serum (data not shown). To investigate whether IL-33 can promote the expression of IL-1β, IL-4, IL-6, and IL-10 in esophageal adenocarcinoma cells, the mRNA levels of the cytokines were detected with or without IL-33 stimulation. The IL-33 stimulation group in OE19 had considerably higher levels of IL-4 and IL-6 mRNA than the control group (Figure 6B). And the level of IL-6 mRNA in the IL-33 stimulation group was considerably higher than in the control group in OE33 (Figure 6C). We further confirmed the increased secretion of IL-6 in the supernatant of OE19 (Figure 6D) and OE33 (Figure 6E) after IL-33 stimulation. However, the content of IL-4 did not change significantly (Figures 6F,G).

## Discussion

Interleukin-33 (IL-33) has been implicated in various inflammatory-driven disorders and malignancies. However, its role in EAC remains unclear. With the progression of GERD to EAC, IL-33 was steadily elevated and released from the nucleus into the cytoplasm and ultimately the extracellular space. IL-33 not only promoted the proliferation, migration, invasion and EMT of esophageal adenocarcinoma cells through ST2, but also promoted the secretion of IL-6. We have revealed for the first time that IL-33 was involved in the inflammatory-driven neoplastic transformation of EAC.

It has been proved that inflammation contributes to tumorigenesis and promotes all stages of tumorigenesis (19), such as chronic gastritis to gastrointestinal metaplasia and eventually leading to tumorigenesis (20), colitis to colonic polyps and eventually leading to colon cancer (21). The occurrence of EAC follows the process of chronic inflammation leading to carcinogenesis. Long-term persistent gastroesophageal reflux results in squamous epithelial injury, while columnar epithelium plays a repair role in place of squamous epithelium to form BE. BE can progress to low-grade and high-grade dysplasia and ultimately to EAC (18, 22). Our previous studies showed that IL-33 mRNA and protein levels were increased in reflux esophagitis patients and participated in the exaggeration of inflammation (8). We use a rat model of gastroesophageal reflux established by anastomosing the jejunum with the esophagus following the established method (18), which can simulate the circumstances of gastroduodenal mixed reflux. This model has been proven to successfully create the whole process from GERD to BE and EAC in previous studies (23, 24). Using this model, we successfully created a gradually progressing pathological stage containing normal, low-grade dysplasia, high-grade dysplasia, and EAC, which provided a good model for reflecting the alteration of IL-33 in this process. We found that the expression of IL-33 further increased gradually with the progress from GERD to EAC in the EAC rat model, suggesting IL-33 was involved in the whole process from esophageal inflammation to tumorigenesis of EAC.

Previous studies have shown that IL-33 is extensively present in the nuclei of endothelial and epithelial cells (23). IL-33 binds to histones and chromatin and acts as a nuclear factor regulating transcription intracellularly (24). Upon cellular damage, IL-33 is released from the cell to bind with its receptor ST2, triggering danger-associated responses and serving as a cellular alarmin (24, 25). After binding with ST2, IL-33 activates downstream signaling molecules, including MyD88 and TRAF6, and finally activates NF-κB, p38, JNK, ERK pathway. Moreover, IL-33 also plays an important role in immune regulation. It can regulate Tregs and Th2 differentiation and function and stimulate immune cells such as dendritic cells, macrophages and mast cells to produce inflammatory cytokines (24, 26, 27). In this study, IHC staining of IL-33 in human EAC tissues showed that IL-33 was mainly expressed in the cytoplasm in EAC and in the nucleus in adjacent normal epithelial cells. This finding was also confirmed in the rat model of EAC, we found IL-33 was mainly expressed in the cytoplasm in esophageal adenocarcinoma cells. Furthermore, we discovered elevated IL-33 levels in the serum of the EAC group in the rat model, implying that IL-33 was released from the nucleus to the cytoplasm and extracellular in the process from inflammation to EAC and played a role in carcinogenesis as a cytokine. Although other studies, such as breast cancer patients (28), gastric cancer patients (29), found that IL-33 in serum was increased, IL-33 was not detected in the serum of patients with normal, esophagitis and EAC, suggesting that it is not suitable to be used as a serum marker for screening EAC.

A variety of tumor-promoting effects of IL-33 have been found in previous studies. IL-33 enhances the migration and invasion of breast cancer (30, 31), gastric cancer (32), colorectal cancer (33) and lung cancer (34). Our research also found IL-33 promoted the proliferation, migration, invasion and EMT of esophageal adenocarcinoma cells through ST2. We further examined the effect of IL-33 on tumor-related cytokines and found that the mRNA expressions of IL-4 and IL-6 were significantly higher in the esophageal adenocarcinoma cells after IL-33 stimulation. IL-33 promotes the secretion of the tumor-promoting cytokine IL-6 by esophageal adenocarcinoma cells. Many previous studies have shown that IL-6 is involved in oncogenesis. It is involved in the proliferation and differentiation of malignant cells, including colorectal cancer (35), breast cancer (36) and pancreatic cancer (37). IL-6 activated EMT in esophageal adenocarcinoma cells and enhanced the migration ability of cells (38, 39). These results showed that IL-33 secreted as a cytokine was involved in the development of EAC by stimulating the proliferation, migration, and invasion of EAC cells through ST2, as well as the secretion of tumor-promoting cytokine IL-6.

In previous studies, it has been shown that IL-33/ST2 can stimulate a variety of immune cell reactions, among which IL-33 stimulation of type 2 innate lymphoid cells promotes macrophage M2 polarization to participate in the maintenance of the tumor microenvironment (40-43). And IL-33 can stimulate the production of cytokines (IL-4, IL-6) and chemokines (CCL2, CCL3, CCL7, CXCL1), which are also involved in the construction of the tumor microenvironment (12, 27, 30, 44, 45). In the next study, we will also explore the role of IL-33 in the tumor microenvironment of esophageal adenocarcinoma.

Our findings reveal that IL-33 expression rises gradually as EAC progresses from GERD through BE to EAC and that IL-33 is released from the nucleus to the cytoplasm and ultimately to the extracellular space. IL-33 is a cytokine involved in the formation of EAC and has been shown to increase the proliferation, migration, and invasion of esophageal adenocarcinoma cells in *in vitro* through ST2.

## Data Availability

The original contributions presented in the study are included in the article/supplementary material, further inquiries can be directed to the corresponding author.

## Abbreviations

BE, Barrett esophagus; EAC, esophageal adenocarcinoma; ELISA, enzyme-linked immunosorbent assay; EMT, epithelial-mesenchymal transition; GERD, gastroesophageal reflux disease; HEECs, normal human esophageal epithelial cells; IHC, immunohistochemistry staining; IL-33, interleukin-33; RT-qPCR, quantitative RT-PCR; sST2, soluble ST2.
